# Reward During Arm Training Improves Impairment and Activity After Stroke: A Randomized Controlled Trial

**DOI:** 10.1177/15459683211062898

**Published:** 2021-12-22

**Authors:** Mario Widmer, Jeremia P. O. Held, Frieder Wittmann, Belen Valladares, Olivier Lambercy, Christian Sturzenegger, Antonella Palla, Kai Lutz, Andreas R. Luft

**Affiliations:** 1 Division of Vascular Neurology and Neurorehabilitation, Department of Neurology, 27243 University Hospital and University of Zurich, Zurich, Switzerland; 2 cereneo Center for Neurology and Rehabilitation, Vitznau, Switzerland; 3 cereneo Advanced Rehabilitation Institute (CARINg), Vitznau, Switzerland; 4 Department of Therapy, 60550 Swiss Paraplegic Centre, Nottwil, Switzerland; 5 Rehabilitation Engineering Laboratory, Department of Health Sciences and Technology, 27219 ETH Zurich, Zurich, Switzerland; 6 386686 Klinik Lengg, Zürcher RehaZentren, Zurich, Switzerland; 7 60527 Bellikon Rehabilitation Clinic, Bellikon, Switzerland; 8 30930 Klinik Wald, Zürcher RehaZentren, Wald, Switzerland; 9 Swiss Concussion Center, 30699 Schulthess Clinic, Zurich, Switzerland; 10 squipe GmbH, Wädenswil, Switzerland

**Keywords:** rehabilitation, stroke, upper extremity, virtual reality, feedback, reward

## Abstract

**Background:**

Learning and learning-related neuroplasticity in motor cortex are potential mechanisms mediating recovery of movement abilities after stroke. These mechanisms depend on dopaminergic projections from midbrain that may encode reward information. Likewise, therapist experience confirms the role of feedback/reward for training efficacy after stroke.

**Objective:**

To test the hypothesis that rehabilitative training can be enhanced by adding performance feedback and monetary rewards.

**Methods:**

This multicentric, assessor-blinded, randomized controlled trial used the ArmeoSenso virtual reality rehabilitation system to train 37 first-ever subacute stroke patients in arm-reaching to moving targets. The rewarded group (n = 19) trained with performance feedback (gameplay) and contingent monetary reward. The control group (n = 18) used the same system without monetary reward and with graphically minimized performance feedback. Primary outcome was the change in the two-dimensional reaching space until the end of the intervention period. Secondary clinical assessments were performed at baseline, after 3 weeks of training (15 1-hour sessions), and at 3 month follow-up. Duration and intensity of the interventions as well as concomitant therapy were comparable between groups.

**Results:**

The two-dimensional reaching space showed an overall improvement but no difference between groups. The rewarded group, however, showed significantly greater improvements from baseline in secondary outcomes assessing arm activity (Box and Block Test at post-training: 6.03±2.95, *P* = .046 and 3 months: 9.66±3.11, *P* = .003; Wolf Motor Function Test [Score] at 3 months: .63±.22, *P* = .007) and arm impairment (Fugl-Meyer Upper Extremity at 3 months: 8.22±3.11, *P* = .011).

**Conclusions:**

Although neutral in its primary outcome, the trial signals a potential facilitating effect of reward on training-mediated improvement of arm paresis.

**Trial registration:**

ClinicalTrials.gov (ID: NCT02257125).

## Introduction

After stroke, 50% of survivors are left with upper extremity impairments,^1,2^ a disability that lowers their health-related quality of life.^3^ Therapies to cure or ameliorate arm impairment are limited in their population efficacy, although some patients respond to therapy or recover spontaneously. Apart from training dose (ie, time spent training), it is unknown what makes training effective and in whom. When training dose is matched, most randomized controlled trials introducing new interventions (eg, robot-assisted therapy)^4^ showed no difference to control being routine care or conventional physical/occupational therapy. Assuming that currently available therapies do not fully exploit the biological recovery potential,^5^ there is urgent need for improvement.

Improvement may be achieved by identifying effective elements of therapy and boosting them. Reward during training may be one such element. In the rat, dopaminergic projections from the midbrain’s ventral tegmental area (VTA) to primary motor cortex (M1) are necessary for successful motor skill learning.^6^ Dopamine in M1 modulates excitability^7^ and enables long-term potentiation of cortico-cortical connections.^8^ Populations of dopaminergic VTA neurons respond to food rewards as well as to the combination of reward and training.^9^ In humans, reward enhances procedural^10^ and motor skill learning^11,12^ and has a positive effect on motor adaptation.^13^ This is mainly the result of improved retention or consolidation.^11–13^ In a functional magnetic resonance imaging study, we demonstrated that adding monetary rewards after good performance leads to better consolidation and higher ventral striatum activation than knowledge of performance alone,^12^ the striatum being a key area of reward processing.^14,15^

While motor skill learning is not the only mechanism mediating movement recovery after stroke, it certainly is an important factor.^16,17^ It therefore seems likely that reward will also affect recovery, as it does skill learning. We thus hypothesized that augmenting reward improves recovery in response to training. Using the ArmeoSenso, a standardized virtual reality-based training system, allowed for delivery of intensive repetitive training of the upper limb^18^ while rewarding features like game scores (linked to a monetary reward), visual and sound special effects of the applied therapy game could be easily manipulated. Here we report a proof-of-concept, assessor-blinded, multicenter randomized controlled trial comparing the effect of enhanced feedback and reward vs unrewarded training matched in time and movement repetitions on arm activity and impairment.

## Materials and Methods

### Study Design

**Figure 1.**
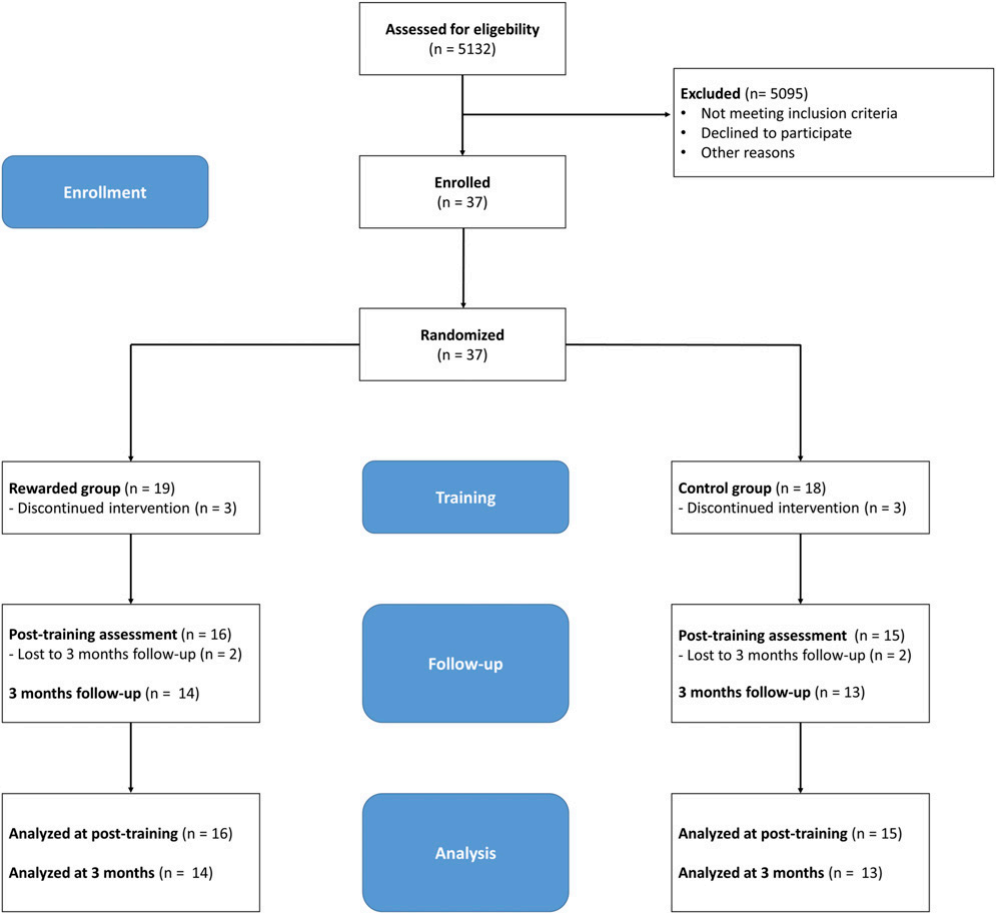
Participant flow through the study. Consolidated Standards of Reporting Trials (CONSORT) flow chart.

ArmeoSenso-Reward was a Swiss national, multicentric, assessor-blinded, parallel-group randomized controlled trial testing the hypothesis that rehabilitative training could be enhanced by reward incentives. Eligible patients were randomized 1:1 to either rewarded or control group using permuted block randomization (blocks of 4) stratified by study center (5 sites).^19^ The randomization procedure was planned and set up by an independent contract research organization (Appletree CI Group, Winterthur, Switzerland). The study protocol including a detailed description of the randomization procedure has been described in a previous publication (see Widmer et al^19^). The study was conducted according to national and international guidelines^20^ and followed the Consolidated Standards of Reporting Trials (CONSORT) statement on randomized trials of non-pharmacological treatment^21^ (see Figure 1 and checklist in Supplementary File 1) and Standard Protocol Items: Recommendations for Interventional Trials (SPIRIT; see Supplementary File 2) guidance for protocol reporting.^22^ Assessors were trained in performing the assessments, blinded to treatment allocation, and patients were unaware of the training characteristics of the other study group. The ArmeoSenso-Reward trial was registered at clinicaltrials.gov (ID: NCT02257125).

### Study Population

This study included subacute ischemic stroke patients (max. 100 days after stroke) that met the following criteria: Minimum age of 18 years, hemiparesis of the arm, ability to lift the paretic arm against gravity with a minimal arm workspace of 20 cm × 20 cm in the horizontal plane (as visually assessed by a member of the study team), ability and willingness to participate, as well as the absence of severe aphasia (ie, patients that were not able to follow 2 stage commands), documented severe depression (medical records), dementia, and hemianopia. Patients were recruited from 5 Swiss stroke rehabilitation centers.

### Interventions

**Figure 2.**
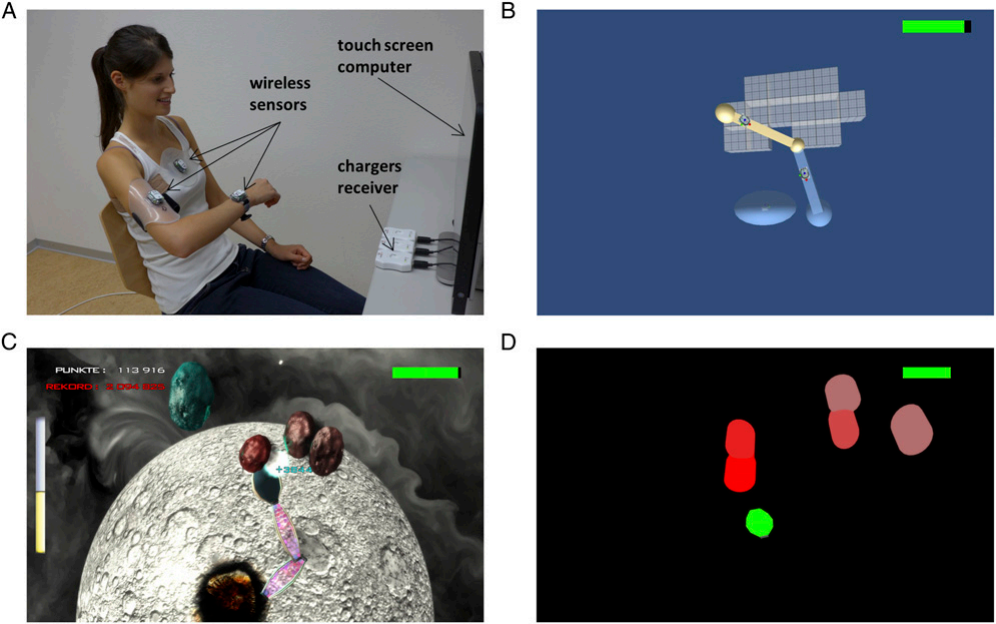
ArmeoSenso-Reward: Device and interventions. (A) Healthy subject using the ArmeoSenso training system. (B) Arm workspace assessment: Gray cubic voxels arranged in the transverse plane reflecting 10 cm × 10 cm active workspace relative to the patient’s trunk. (C) Rewarded training using the METEORS game: The hand of the virtual arm was used to catch the falling meteors before they crash onto the planet. If caught, the meteor exploded (visual and auditory feedback), and a score appeared (visual feedback). The earlier the meteor was caught, the higher was the produced score. If missed, the planet got damaged (note the impact crater (visual and auditory feedback)). Monetary rewards were given for each completed level. Patients could win up to 1 Swiss Franc (CHF), if they succeeded, but .1 CHF was deducted for every missed meteor. As a new level could be started approximately every 3 minutes, a maximum of 20 CHF (approx. 20 US-Dollars) could be won per training session in case of an uninterrupted winning streak. Summary statistics and monetary rewards were displayed visually after successfully completing a level. (D) Control game. The virtual hand was a green decagon that could be used to touch the pill-shaped, single-colored targets dropping in from the top of the screen, which then disappeared with a delay of 1 s without producing visual or sound effects and without producing a score. For more details, see Widmer et al.^19^

The ArmeoSenso arm rehabilitation system combines motion capturing via wearable inertial measurement units (IMUs)^23^ and a therapy game consisting of fast 3-dimensional target reaching movements (Figure 2(A)).^18,24^ Three wireless IMUs (MotionPod 3, Movea SA, Grenoble, France) are fixed to the functionally impaired lower and upper arm as well as the trunk. Note that what we refer to as “ArmeoSenso” in this work is a research prototype of the Armeo®Senso product (Hocoma AG, Volketswil, Switzerland), using different hardware and custom-developed software for therapy and assessments.

Both groups, rewarded and control, trained with modified versions of the ArmeoSenso “METEORS” game^18,24^: The rewarded group with a version including knowledge of performance feedback (ie, explosions, game scores, and hall of fame), contingent monetary rewards, an emotionally involving game theme (ie, subjects were instructed to protect their planet from being destroyed by a meteor shower) with graphical and sound effects (Figure 2(C)), and the control group with a version lacking these motivators (Figure 2(D), more details can be found in the caption of Figure 2 and in Widmer et al^19^). Although the 2 versions differed markedly in terms of their appearance, they shared the underlying game mechanics and required the same type and amount of movement by the subject, with automatic difficulty adaptation (described in detail in Widmer et al^19^). That is, in both, a virtual “hand” which matched the movement of the subject’s real hand was used to catch objects that dropped down from the top of the screen. Notably, the trunk sensor was used to estimate the orientation of the trunk. Only arm movements relative to the trunk were detected by the system. The targets were placed within or at the border of the patient’s virtual 3D workspace, which was continuously estimated and updated in the background using a voxel-based model.^24^ The number of objects caught by the virtual hand was taken as a surrogate for arm activity during a session (training intensity). Both groups trained under supervision by a therapist for 1 hour per day, 5 days a week for 3 weeks in addition to standard therapy. Supervising therapists ensured proper setup but were otherwise instructed to remain passive. Patients of the rewarded group were paid by bank transfer after completing the intervention. Payment was initiated by the supervising therapist after the last training.

### Outcomes

The primary outcome of this trial was the workspace of the impaired arm in the horizontal plane, measured using an assessment integrated into the ArmeoSenso platform.^18^ Subjects were instructed to actively reach out as far as possible with their impaired arm forward, backward and sideways to explore the entire arm workspace. The attained arm workspace (relative to the patient’s trunk as described above) projected onto the transverse plane and hence independent from the shoulder flexion angle was computed as the number of reached 10 cm × 10 cm squares displayed to the patient as cubic voxels (Figure 2(B)). This assessment has been shown to correlate significantly with the Fugl-Meyer Assessment–Upper Extremity (FMA-UE).^18^ Here, it was conducted immediately before and after every therapy session together with a pointing task. For the pointing task, the average time needed to reach each one of 8 targets arranged in 2 semicircles at fixed positions (ie, identical for each participant) appearing sequentially in the transversal plane in front of the subject was measured. For each trial, the virtual hand represented as crosshair had to be moved from a circular starting area right in front of the subject to the displayed target circle. The sequence for the presentation of the targets was randomly selected by the system. If the target could not be reached within a maximum time of 8 seconds, this maximum time was registered, and the next target was presented.

Clinical scores were collected at 3 timepoints: baseline, post-training, and 3 month follow-up. Arm motor impairment was assessed using the FMA-UE^25^ and arm activity using the Wolf Motor Function Test (WMFT)^26^ and the Box and Block Test (BBT).^27^ While the WMFT was used to test a broader variety of functional tasks, the BBT was included because with its repetitive characteristic it was assumed to be closer to what was trained during our intervention. Motor Activity Log 14 (MAL-14) including amount of use (AOU) and quality of movement (QOM) subscales was used for self-reported movement ability,^28^ the Barthel Index (BI) as a measure of independence in daily living,^29^ and the National Institutes of Health Stroke Scale (NIHSS) as a measure of stroke severity. Global disability was assessed using the modified Rankin Scale (mRS).^30^

Finally, patients filled in a short motivation questionnaire after each training session. Ten questions (5 positively and 5 negatively formulated) put together by the study team evaluated the subjective appraisal of the training, each question rated on a five-point Likert scale. Negative questions have been reverse scored and an average score was calculated. Psychometric properties of this scale have been calculated with data from the current patient sample as internal consistency determined at day 1 (Cronbach’s alpha = .839) and retest reliability of the individual average scores from day 1 with day 2 (Spearman’s rho = .746).

Adverse events (AEs) were documented from baseline assessment to the end of the trial. AEs expected to occur were skeletal or muscular pain and fatigue indicating a syndrome of overuse.

In addition to the outcomes described above, demographics, comorbidities, suspected cognitive impairment (Mini-Mental State Examination), and concomitant therapy were recorded.

### Statistical Analysis

Our primary analysis was an intention-to-treat analysis comparing the 2 groups. For the primary outcome, a 2-sample *t*-test comparing the mean change in voxel workspace assessment between the 2 groups was used. Based on a 2-sided α level set at .05 and a power of 80%, we have calculated the sample size required to detect an estimated group difference of 20% (4.8 voxels) and a standard deviation of 7 voxels in the primary outcome measure to be 35 per group.^19^ Assuming a drop-out rate of 5% in analogy with a previous trial,^18^ we planned to include 37 subjects in each group.

For secondary clinical outcomes, each outcome measure (except the mRS) was analyzed using the same framework and model structure. Specifically, linear mixed-effect models (LMMs) implemented in the lme4 package in R were used to detect changes of the clinical scores over time.^31^ Timepoint (baseline, post-training and 3 months; reference: baseline) and group (rewarded and control; reference: control) were considered fixed factors. A subject-specific random intercept accounted for within-subject correlation across timepoints. This model structure estimates the mean change in outcome value from baseline to each timepoint as well as the difference in this change over time comparing groups, using all available subject data at each visit.

For the analysis of the mRS data, a cumulative link mixed model was fitted with the clmm function implemented in the ordinal package in R using the same model structure as described above.

Data collected during the intervention period, that is, voxel workspace assessment, pointing task, and motivation questionnaires, was also explored using LMMs in which the training day was treated as a categorical predictor with 15 levels (reference: Training 1) and study group was a categorical variable with 2 levels (reference: Control). Here, the subject-specific random intercept accounted for within-subject correlation across training days.

For all the LMMs described above, normal distribution of residuals of the resulting model was visually confirmed using normality plots.

Finally, 2-sample *t*-tests or, in case of non-normality, Mann–Whitney *U* tests were performed to directly compare group characteristics (eg, baseline characteristics). Normal distribution of the dependent variable was assessed using the Shapiro–Wilk test.

Statistical significance was based on a 2-sided *P* value threshold of .05.

### Study Approval

The ArmeoSenso-Reward study was approved by the responsible ethics committees “Ethikkommission Nordwest- und Zentralschweiz” and the “Kantonale Ethikkommission Zürich” (LU2013-079 and PB_2016-01804), for each participating rehabilitation clinic, and the Swiss Agency for Therapeutic Products (Swissmedic: 2014-MD-0033). All subjects gave written informed consent in accordance with the Declaration of Helsinki.

## Data Availability

The data that support the findings of this study are available from the corresponding author upon reasonable request.

## Results

Thirty-seven patients were enrolled between January 2015 and December 2019. Due to AEs occurring in similar frequency in both groups and an interruption of recruitment due to the COVID-19 pandemic restrictions, we terminated the study after inclusion of 50% of the intended patients. All available data of patients that were randomized were included in the intention-to-treat analysis. Table 1 summarizes baseline characteristics of the cohort. For a schematic overview of the participant flow through the study, see Figure 1.

**Table 1.** Baseline characteristics.

	Overall (n = 37)	Rewarded (n = 19)	Control (n = 18)	*P* value
Days post-stroke, mean (SD)	38.22 (24.36)	35.32 (28.16)	41.28 (19.96)	*.19*
Age, mean (SD)	64.46 (13.42)	63.05 (14.82)	65.94 (12.02)	*.52*
Gender female, # (%)	8 (21.62%)	5 (26.32%)	3 (16.67%)	*.41*
Right affected, # (%)	13 (35.14%)	6 (31.58%)	7 (38.89%)	*.68*
Dominant affected, # (%)	14 (37.84%)	7 (36.84%)	7 (38.89)	*1.00*
BMI, mean (SD)	26.07 (4.40)	26.91 (4.72)	25.18 (3.97)	*.24*
MMST, median (IQR)	28.0 (4.0)	28 (2.0)	28.5 (6.25)	*.79*
mRS, median (IQR)	3.0 (.0)	3.0 (1.0)	3.0 (.0)	*.31*
NIHSS, median (IQR)	5.0 (2.0)	4.0 (2.5)	5.0 (2.75)	*.49*
Barthel Index, median (IQR)	17.0 (4.0)	17.0 (2.5)	17.5 (3.75)	.56
FMA-UE, mean (SD)	32.81 (10.86)	32.05 (12.04)	33.61 (9.75)	.67
WMFT Score, mean (SD)	3.09 (.88)	3.05 (1.03)	3.14 (.72)	.77
WMFT Time, median (IQR) [s]	11.83 (22.13)	11.83 (36.30)	11.50 (19.87)	.78
Box and Block Test, mean (SD)	17.35 (14.67)	20.05 (16.97)	14.50 (11.60)	.25

Baseline characteristics of all the patients that have been randomized to either the rewarded or the control group.

No significant difference in any of the reported metrics was observed, as revealed by 2-sample *t*-tests or Mann–Whitney *U* tests.

BMI: body mass index; MMST: Mini-Mental State Examination; mRS: modified Rankin Scale; NIHSS: National Institutes of Health Stroke Scale; FMA-UE: Fugl-Meyer Assessment–Upper Extremity; WMFT: Wolf Motor Function Test; SD: standard deviation; IQR: interquartile range.

### Primary Outcome

**Figure 3.**
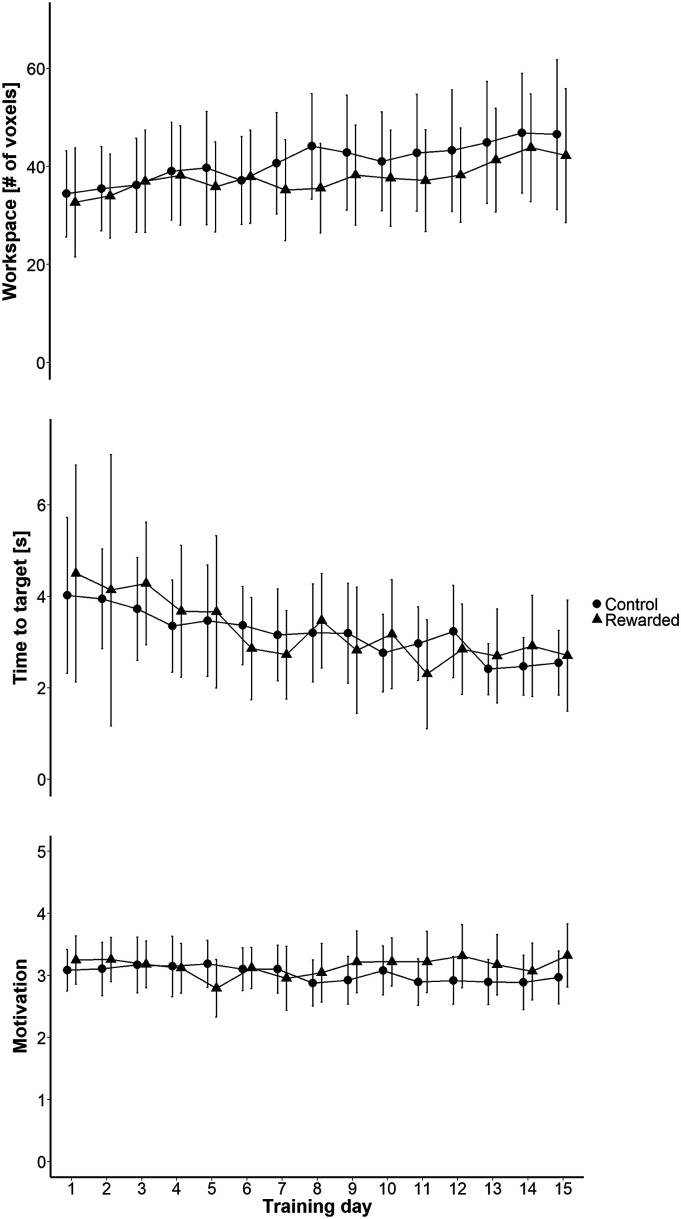
ArmeoSenso integrated assessments and motivation. Results from the ArmeoSenso Workspace (primary outcome) and Pointing Task Assessment, as well as from the motivation questionnaire. These outcomes were assessed each day during the training period. Data is presented as mean and confidence interval.

In the predefined primary outcome measure, the change of the arm workspace assessment over the intervention period, no difference between groups was found (*t* (380.21) = −.73, *P* = .47, Figure 3). Groups were similar at Training 1 (*t* (26.46) = −.27, *P* = .79) and improved significantly over the training period (*F* (14, 393.47) = 5.70, *P* < .001). Neither the main effect “group” (*t* (33.15) = −.56, *P* = .58) nor the interaction term “training session × group” (χ^2^ (14) = 12.71, *P* = .55) significantly predicted the number of voxels reached in the workspace assessment. However, taking all patients together, they continuously improved in this assessment showing significant change at training day 5 and 7 to 15 (when compared to Training 1).

### Secondary Outcomes

**Figure 4.**
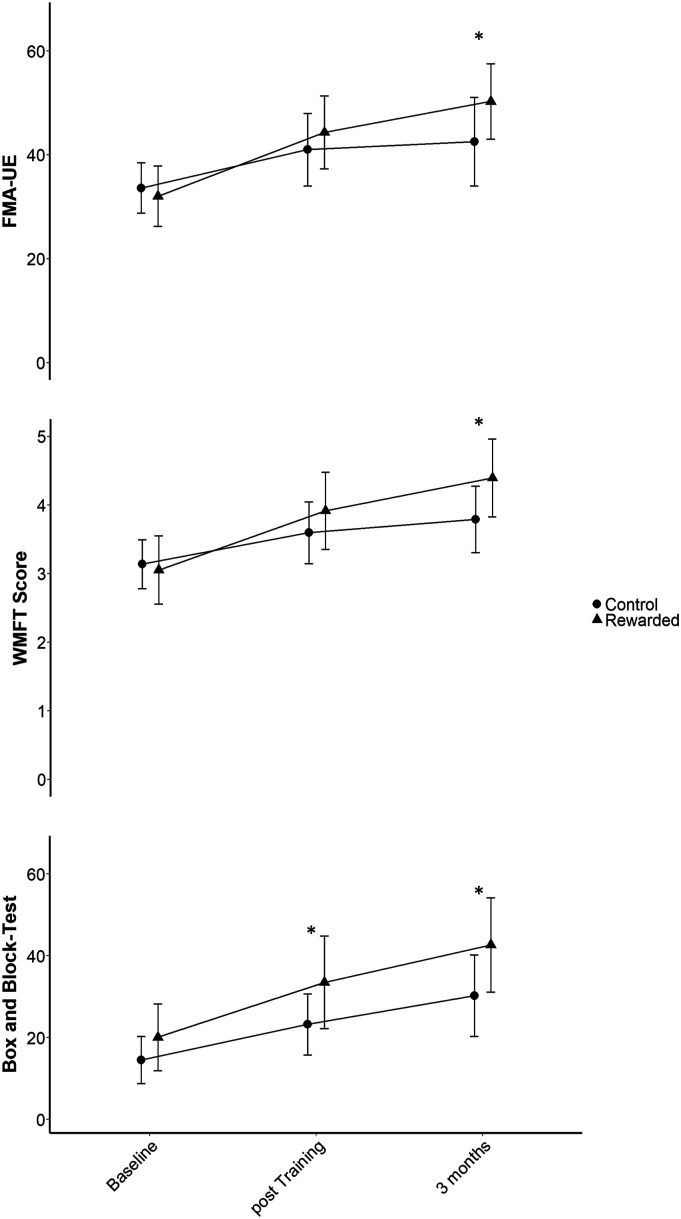
Secondary clinical outcomes. Fugl-Meyer Assessment–Upper Extremity (FMA-UE), Wolf Motor Function Test (WMFT) and Box and Block Test showing significant between-group differences in change from baseline. Data is presented as mean and confidence interval.

Overall, patients improved their FMA-UE score from baseline to post-training (mixed model estimate: 9.24 (standard error (SE) 1.53), *t* (58.52) = 6.03, *P* < .001) and continued doing so until 3 months (13.81 (SE 1.62), *t* (58.86) = 8.56, *P* < .001). However, introducing the “time × group” interaction significantly enhanced the model (χ^2^ (2) = 7.12, *P* = .028). The improvement in FMA-UE was greater in the rewarded group at post-training (4.06 (SE 2.96), *t* (56.47) = 1.37, *P* = .18) and significantly so at 3 months (8.22 (SE 3.11), *t* (56.78) = 2.64, *P* = .011; Figure 4). The minimal clinically important difference (MCID) for the FM-UE is considered to be approximately 10% of the maximum score, or 6.6 points.^25^ Hence, the estimated difference in change of 8.22 points from baseline to 3 months post-intervention was clinically important.

Over the course of the study, patients improved in the average WMFT score (post-training: .62 (SE .11), *t* (56.75)=5.66, *P* < .001; 3 months: .97 (SE .12), *t* (57.13)=8.31, P<.001) and time (post-training [s]: −8.99 (SE 2.81), *t* (53.99) = −3.20, *P* = .002; 3 months [s]: −15.02 (SE 3.00), *t* (54.55) = −5.01, *P* < .001). In the additive model, the fixed effect “group” was neither a significant predictor of the score (*t* (34.64) = .63, *P* = .53) nor of the time needed to complete the exercises (*t* (31.33) = .29, *P* = .77). Introducing the “time × group” interaction, however, improved the model fit for the WMFT score (χ^2^ (2) = 8.22, *P* = .016), but not for WMFT time (χ^2^ (2) = .90, *P* = .64). Between-group differences in change of the WMFT score with respect to baseline showed a positive trend at post-training (.36 (SE .21), *t* (54.67) = 1.71, *P* = .093) and significance at 3 months (*t* (55.00) = 2.82, *P* = .0067) with an estimated difference of .63 (SE .22) points average change per item from baseline (Figure 4).

Number of blocks achieved in the BBT with the impaired side increased from baseline to post-training (10.91 (SE 1.58), *t* (57.56) = 6.90, *P* < .001) and then further until 3 months (19.20 (SE 1.67), *t* (57.73) = 11.52, *P* < .001). The fixed effect “group” showed a trend in the additive model (*t* (35.23) = 1.85, *P* = .072). However, introducing the “time × group” interaction term significantly improved the model fit (χ^2^ (2) = 9.85, *P* = .0073). Scores of the rewarded group improved more until post-training (6.03 (SE 2.95) blocks, *t* (55.30) = 2.04, *P* = .046) and the gap widened even further until 3 months (*t* (55.44) = 3.11, *P* = .0030), reaching an estimated between-group difference of 9.66 (SE 3.11) blocks change from baseline (Figure 4). This clearly exceeds the MCID of 5.5 blocks/minute for the BBT.^32^

MAL-14 subscales AOU and QOM were similar between groups at baseline (both *P* > .8) and then improved over time (both P<.001). No significant main effect of the group allocation (*t* (35.50) = .90, *P* = .37 and *t* (35.30) = .73, *P* = .47, respectively) or “time × group” interaction (χ^2^ (2) = .40, *P* = .82 and χ^2^ (2) = .95, *P* = .62, respectively) was observed (Table 2).

**Table 2.** Development of stroke severity (NIHSS), ability to perform activities of daily living (Barthel Index), level of functional independence (mRS), and self-reported movement ability (MAL-14) over the study period.

	NIHSS, mean (SD)		Barthel Index, mean (SD)		mRS, median (IQR)		MAL-14: AOU, mean (SD)		MAL-14: QOM, mean (SD)	
	Rewarded	Control	Rewarded	Control	Rewarded	Control	Rewarded	Control	Rewarded	Control
Baseline	4.26 (2.05)	4.56 (1.69)	16.00 (3.65)	16.89 (2.52)	3.0 (1.0)	3.0 (.0)	1.14 (1.44)	1.26 (.68)	1.32 (1.12)	1.16 (.57)
Post-training	2.50 (1.75)	2.73 (1.28)	18.31 (2.30)	18.93 (1.16)	3.0 (.0)	3.0 (.0)	2.23 (2.48)	1.83 (1.20)	2.26 (1.21)	1.97 (1.06)
3 months	2.29 (1.54)	2.15 (1.46)	18.93 (2.09)	19.15 (1.14)	2.0 (1.0)	3.0 (1.0)	2.56 (1.70)	2.09 (2.09)	2.58 (1.28)	2.15 (1.30)

NIHSS: National Institutes of Health Stroke Scale; mRS: modified Rankin Scale; MAL-14: Motor Activity Log 14; AOU: amount of use; QOM: quality of movement; SD: standard deviation; IQR: interquartile range.

With n = 19, n = 16, and n = 14 for the rewarded group at baseline, post-training, and 3 month follow-up, respectively, and n = 18, n = 15, and n = 13 for the control group at baseline, post-training, and 3 month follow-up, respectively.

The NIHSS, BI, and mRS, similarly, improved over time without any differences between groups (Table 2).

For the pointing task assessment (Figure 3), groups performed similarly at Training 1 (*t* (9.69) = .42, *P* = .69). Overall, patients improved over time showing significant change from the third training onwards when compared to the first training. However, neither the fixed effect “group” (*t* (25.06) = .22, *P* = .83) nor the interaction term “training session × group” (χ^2^ (14) = 15.11, *P* = .37) significantly predicted the average time that was needed to reach the targets.

Results from the motivation questionnaire implied that the subjective appraisal of the training remained relatively stable over the 15 trainings (*F* (14, 422.76) = 1.32, *P* = .19) with no overall difference between groups (*F* (1, 33.22) = .63, *P* = .43). However, there was a trend towards a “training session × group” interaction (χ^2^ (14) = 23.60, *P* = .051) as an indication for a different development of motivation to train over time, reflecting the sudden drop of scores in the rewarded group at training 5, but also the constantly higher scores in the same group towards the end of the 3 weeks training period (Figure 3).

The rewarded group earned a median of 131.70 (interquartile range (IQR) 66.90) Swiss Francs over the course of the intervention. No financial compensation was paid to the control group. Yet, the number of targets caught successfully was not different between groups (6680 successful reaches, IQR 10071 vs 6718 successful reaches, IQR 6552.5, *W* = 179, *P* = .40 for the rewarded and the control group, respectively). An overview of the concomitant standard therapy separated into physical therapy, occupational therapy, medical training therapy, neuropsychology, speech therapy, and others can be found in Supplementary File 3. No significant differences in total therapy time or any of the therapy modalities were observed, as revealed by Mann–Whitney *U* tests.

### Adverse Events

No related serious adverse events (SAEs) have occurred during the course of the trial. Overall, therapy-related AEs have been reported for 11 patients (29.7%). In the rewarded group, 3 patients had a therapy-related AE (fatigue in the shoulder after the training and 2 times shoulder pain). The fatigue led to the discontinuation of the study. Notably, none of the AEs in the rewarded group were associated with specific features of the rewarding version of the therapy system. In the control group, intervention-related AEs were reported for 8 patients. Shoulder pain was the most frequent AE (6 patients). Redness and marks on the skin from the Velcro straps (1 patient), as well as fatigue (1 patient), were also reported. Two control subjects chose to discontinue the intervention.

## Discussion

This is the first randomized clinical trial to evaluate the effect of enhanced feedback and reward on arm rehabilitative training following stroke. Through a sensor-based virtual reality training system, the ArmeoSenso, patients were trained to lift their arm against gravity and were required by the game mechanics to increase their range of motion in order to succeed in the task. The rewarded group trained with performance feedback (gameplay) and contingent monetary reward. The control group used the same system but without monetary reward and with graphically minimized performance feedback. Patients improved their active workspace in the transverse plane, but with no difference between groups. Clinical scores, however, showed statistically significant greater improvements for the rewarded group in the BBT at post-training and in the WMFT score, the FMA-UE, and the BBT at 3 month follow-up, exceeding a MCID for the latter 2. Although the trial is neutral in its primary endpoint (workspace as measured by number of 10 cm × 10 cm voxels in the transversal plane), it shows a positive signal for reward to potentially facilitate arm training after stroke.

This trial supports the clinical experience that positive feedback during training motivates patients, which may lead to better results. But our findings suggest that this effect is not caused by longer or more intense training enabled by better motivation because both groups in our trial trained, by design, with the same duration and intensity as measured in the number of successful reaches. The effect of reward, therefore, seems to be a direct effect on training-mediated improvement in activity and impairment (as far as the FMA-UE captures impairment).^25^ In a motor learning study with healthy young subjects, we have shown that the consolidation/retention of a skilled motor task is more effective if the task was trained in the presence of reward,^12^ thereby corroborating other studies that have investigated the influence of reward on procedural learning,^10^ motor skill learning,^11^ and motor adaptation.^13^ However, in our experiment, improved skill learning was associated with higher activation of the ventral striatum. The striatum is a key locus of reward processing,^14^ and its activity was shown to be increased by both intrinsic and extrinsic reward.^15^ Being a brain structure that receives substantial dopaminergic input from the midbrain, ventral striatal activity can be taken as a surrogate marker for the activity of dopaminergic neurons in the substantia nigra/ventral tegmental area.^33^

A direct effect of reward on learning and M1 plasticity is in line with our results from healthy rats learning a skilled reaching task: improvement depends on intact dopaminergic projections from the midbrain to M1. These projections enable synaptic plasticity in M1.^6,8^ If these projections indeed transmit reward information—which is likely but has not been definitively demonstrated yet—they could explain the findings of our trial.

Interestingly, the effects of the intervention on secondary clinical scores persisted after the training ended. In fact, the positive effects of the enhanced feedback and reward seemed to continue leading to the largest difference between groups at 3 month follow-up. This is in line with findings from an experiment in healthy subjects, where the study group that was training a skilled motor task under rewarded conditions showed significant offline (post-training) improvements, whereas neutral and punished groups did not. Moreover, in their experiment, the rewarded group retained the gains 1 month after the training, while the other 2 groups experienced significant forgetting.^11^ The authors speculated that the cerebellum could contribute to error-based online learning, whereas the striatum and neocortex may become engaged in later stages and for long-term retention under rewarded conditions.^34,35^ The higher striatal activation leading to better consolidation of the trained motor skill observed in the rewarded group in our aforementioned experiment further supports this hypothesis.^12^ However, whether it really was the stimulation of the reward system or, for example, just the better use of the provided feedback (as speculated in a study investigating the effect of virtual reality in chronic stroke)^36^ in the rewarded group leading to the observed improvements needs to be confirmed in future studies.

Improved overall motivation to train may be another factor contributing to these lasting effects observed in clinical scores in the rewarded group.^37^ An individual’s motivation to perform a specific exercise or activity is determined by the subjective benefit and the subjective cost of the activity.^38^ Here, by implementing rewarding features into rehabilitative training, we aimed at manipulating the subjective benefit of therapy in order to increase our subjects’ motivation to train.^37^ Over the course of the intervention period, patients subjectively rated their motivation for the ArmeoSenso training on a daily basis using a questionnaire (Figure 3). The score remained relatively stable over time, which indicates that the motivation to train remained good, also towards the end of the intervention. However, our reward intervention did not significantly improve this measure (although there was a trend). The use of a self-created (and not validated) motivation questionnaire limits the informative value of this analysis. May well be that our tool was not sensitive enough to appropriately capture differences in the motivational status of the 2 groups. Increased motivation could have transferred to daily life and could have led to a more extensive use of the affected arm during activities of daily living until follow-up. Routine therapy which continued during this period was comparable between groups and can therefore not explain our findings.

In a previous study, we investigated processing of rewarding feedback information in subacute stroke patients.^39^ Interestingly, we found marked hypoactivation of the ventral striatum in stroke patients as compared with healthy age-matched controls when rewarding feedback was given after successfully completing a motor task. This finding matches evidence for atrophy in dopaminergic brain regions after stroke.^40^ However, in the present study, our intervention applied a broad variety of rewarding performance feedback like visual and sound effects (eg, explosions). Moreover, we even used money as a strong universal reward. Hence, although possibly deficient when compared to healthy subjects, reward centers of the rewarded group of the present study might still have been more strongly activated during the training as it was the case for the control group, therefore leading to the between-group differences observed in the development of the clinical scales. Further studies are now needed to disentangle which aspects of our reward intervention are responsible for the beneficial effect described here.

Among all secondary outcome measures, the greatest between-group difference was observed for the BBT. This finding is noticeable, considering that the ArmeoSenso trains proximal arm function. Improvements could reflect a time gain following easier movements against gravity (lifting blocks) and throughout the workspace (transferring blocks), as trained with the ArmeoSenso, allowing for more blocks to be moved from one side to the other. However, the BBT also requires the hand. Our findings may therefore be interpreted as evidence for the transfer of training contents to non-trained movements, which may be facilitated by means of additional reward. Alternatively, improved proximal arm function may allow patients to better engage in situations where they can use/train distal arm function in daily life leading to the observed differences in the BBT at post-training and follow-up. The transfer, however, did not go as far as improving activities of daily living or independence in daily life as suggested by the absence of group effects on BI, MAL-14, and mRS. We did not expect to improve these measures because to do so, a more holistic training also targeting mobility and cognitive function is often necessary.

The effects of adding feedback and reward on clinical outcome measures proved to be significant despite the limited number of patients. This points to a robust effect (as confirmed by the reported mixed model estimates for between-group differences), which is uncommon in rehabilitation research. However, with regard to the interpretation of the primary outcome, it is an evident limitation that this study had to be terminated after inclusion of 50% of the anticipated patients. The main reasons for study termination were COVID-19 pandemic restrictions and therapy-related AEs occurring in both groups (rewarded: 3, control: 8). The patients developed shoulder pain which could have been caused by the arm training which promoted ballistic movements with the paretic arm to reach the targets on the screen. Although shoulder pain is frequent in hemiparetic stroke patients (10-22%),^41^ the temporal relationship with the training raised concerns and we terminated the trial prematurely.

There was a non-significant (*P* = .19) trend towards an earlier inclusion (35 days vs 41 days) with a larger standard deviation for the rewarded when compared to the control group (Table 1) that needs to be acknowledged. An imbalance that is likely owed to the small sample size as discussed above.

A further limitation is that our primary outcome lacks information on clinimetric properties like MCID. We chose a primary outcome that is close to what is actually being trained, that is, arm workspace. Workspace assessments have been widely used to assess the arm function of stroke patients and show a good correlation with standard clinical scales.^18,24,42,43^ Not surprisingly, the workspace increased in both groups, as also both groups improved in clinical scales. But the workspace assessment did not reflect the differences observed in those clinical scales between groups. Possibly, ArmeoSenso training has stronger effects on motor coordination and visuomotor control rather than workspace. Hence, workspace improvements were small and did not capture between-group differences in therapy progression.

The trial was neutral in its predefined primary outcome. Nevertheless, this is the first randomized clinical trial to show a positive effect of a combination of enhanced feedback and reward on several established clinical scales. Clinical scores for arm activity and impairment provide a promising signal in favor of rewarded training. Future trials need to confirm this signal.

## Supplemental Material

sj-pdf-1-nnr-10.1177_15459683211062898 – Reward During Arm Training Improves Impairment and Activity After Stroke: A Randomized Controlled Trial Click here for additional data file. Supplemental Material, sj-pdf-1-nnr-10.1177_15459683211062898 for Reward During Arm Training Improves Impairment and Activity After Stroke: A Randomized Controlled Trial by Mario Widmer, Jeremia P. O. Held, Wittmann Frieder, Belen Valladares, Olivier Lambercy, Christian Sturzenegger, Antonella Palla, Kai Lutz and Andreas R. Luft in Neurorehabilitation and Neural Repair

sj-tif-2-nnr-10.1177_15459683211062898 – Reward During Arm Training Improves Impairment and Activity After Stroke: A Randomized Controlled Trial Click here for additional data file. Supplemental Material, sj-tif-2-nnr-10.1177_15459683211062898 for Reward During Arm Training Improves Impairment and Activity After Stroke: A Randomized Controlled Trial by Mario Widmer, Jeremia P. O. Held, Wittmann Frieder, Belen Valladares, Olivier Lambercy, Christian Sturzenegger, Antonella Palla, Kai Lutz and Andreas R. Luft in Neurorehabilitation and Neural Repair

sj-pdf-3-nnr-10.1177_15459683211062898 – Reward During Arm Training Improves Impairment and Activity After Stroke: A Randomized Controlled Trial Click here for additional data file. Supplemental Material, sj-pdf-3-nnr-10.1177_15459683211062898 for Reward During Arm Training Improves Impairment and Activity After Stroke: A Randomized Controlled Trial by Mario Widmer, Jeremia P. O. Held, Wittmann Frieder, Belen Valladares, Olivier Lambercy, Christian Sturzenegger, Antonella Palla, Kai Lutz and Andreas R. Luft in Neurorehabilitation and Neural Repair
