# Supplementary figures and images for: Reward During Arm Training Improves Impairment and Activity After Stroke: A Randomized Controlled Trial
Source: Neurorehabil Neural Repair. 2021 Dec 22;36(2):140–50. doi: 10.1177/15459683211062898 (PMC8796156; doi:10.1177/15459683211062898)

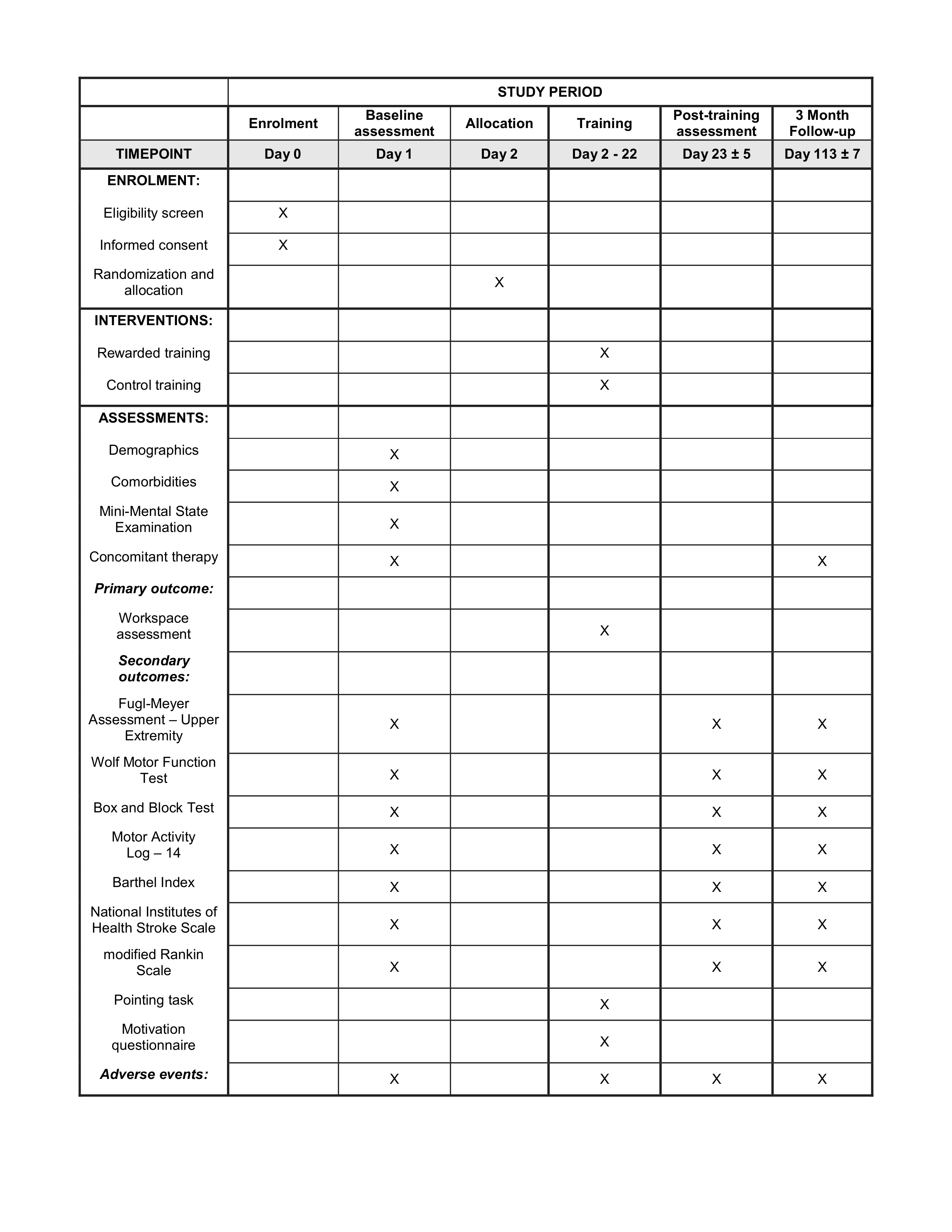

Supplement: sj-tif-2-nnr-10.1177_15459683211062898 – Reward During Arm Training Improves Impairment and Activity After Stroke: A Randomized Controlled Trial [file sj-tif-2-nnr-10.1177_15459683211062898.tif]
