# Supplementary material for: Reward During Arm Training Improves Impairment and Activity After Stroke: A Randomized Controlled Trial
Source: Neurorehabil Neural Repair. 2021 Dec 22;36(2):140–50. doi: 10.1177/15459683211062898 (PMC8796156; doi:10.1177/15459683211062898)
Supplement: sj-pdf-3-nnr-10.1177_15459683211062898 – Reward During Arm Training Improves Impairment and Activity After Stroke: A Randomized Controlled Trial [file sj-pdf-3-nnr-10.1177_15459683211062898.pdf]

Supplementary File 3. **Concomitant standard therapy.**

| | During intervention | | Until 3 months follow-up | |
|---|---|---|---|---|
| | Rewarded (n=19) | Control (n=18) | Rewarded (n=14) | Control (n=13) |
| OT | 16: 5.0 (1.3) | 13: 3.3 (5.4) | 12: 1.5 (0.7) | 12: 1.8 (1.1) |
| PT | 17: 5.0 (2.6) | 15: 5.5 (4.9) | 10: 1.5 (0.8) | 11: 1.5 (1.1) |
| MTT | 8: 2.2 (1.5) | 8: 2.5 (3.6) | 2: 0.8 (0.3) | 4: 2.0 (0.1) |
| Neuropsychology | 10: 3.4 (1.9) | 12: 3.0 (2.0) | 3: 2.0 (0.2) | 3: 1.0 (0.3) |
| Speech therapy | 11: 3.0 (2.4) | 11: 1.7 (1.2) | 1: 0.8 (0) | 2: 2.0 (0) |
| Other | 7: 1.5 (0.6) | 3: 0.6 (0.3) | - | - |
| Total | 17: 14.8 (4.1) | 15: 14.0 (5.3) | 13: 3.2 (0.8) | 12: 4.0 (2.9) |

Concomitant therapy during the intervention period and up to 3 months after the experimental intervention. Data is presented as number of patients receiving that type of therapy: hours per week as median (interquartile range) for patients that received this type of therapy. No significant differences in total therapy time or any of the therapy modalities were observed, as revealed by Mann-Whitney *U* tests. OT: occupational therapy, PT: physical therapy, MTT: medical training therapy.
